# Genetic diversity and drug resistance of *Mycobacterium tuberculosis* in Yunnan, China

**DOI:** 10.1002/jcla.22884

**Published:** 2019-03-21

**Authors:** Daoqun Li, Yuzhu Song, Pengpeng Yang, Xiaofei Li, A‐Mei Zhang, Xueshan Xia

**Affiliations:** ^1^ Faculty of Environmental Science and Engineering Kunming University of Science and Technology Kunming China; ^2^ Faculty of Life Science and Technology Kunming University of Science and Technology Kunming China; ^3^ Department of Clinical Laboratory The Third People’s Hospital of Kunming City Kunming China

**Keywords:** 24‐locus MIRU‐VNTR, Beijing family, drug resistance, *Mycobacterium tuberculosis*, Yunnan

## Abstract

**Background:**

China is a country with high burden of tuberculosis (TB), especially drug‐resistant TB (DR‐TB), which is still a serious health problem in Yunnan Province. *Mycobacterium tuberculosis* (MTB) is the pathogenic microorganism of TB. The epidemiological characteristics of MTB strains in local areas need to be described.

**Methods:**

A total of 430 clinical MTB isolates were collected from Yunnan Province and genotyped through the method of 24‐locus mycobacterial interspersed repetitive unit‐variable number tandem DNA repeats (MIRU‐VNTR).

**Results:**

The genotypes of the 24 loci showed abundantly genetic diversity, and allelic diversity index (*h*) of these loci varied from 0.012 to 0.817. Among the 430 strains, 30 clusters and 370 unique genotypes were identified. Beijing family was the predominant lineage (70.47%) in Yunnan MTB strains, and the other lineages contained T family (5.81%), MANU2 (0.70%), LAM (3.26%), CAS (0.23%), New‐1 (8.37%), and some unknown clades (11.16%). A total of 74 TB strains were identified as drug resistance through drug susceptibility testing (DST), including 38 multidrug‐resistant TB (MDR‐TB) and 36 single‐drug‐resistant TB (SDR‐TB). The frequency of MDR‐TB strains was significantly higher in Beijing family (10.89%) than that in non‐Beijing family (3.94%, *P = *0.032).

**Conclusions:**

Although MTB strains showed high genetic diversity in Yunnan, China, the Beijing family was still the dominant strain. A high frequency of MDR‐TB strains was recorded in the Beijing family.

## 1 INTRODUCTION

Currently, tuberculosis (TB) is still the major pathogen of infectious diseases and the 9th cause of death worldwide.^1^ The lung is mostly infected by *Mycobacterium tuberculosis* (MTB), whereas other tissues are rarely involved. According to the “Global tuberculosis report 2017,”^2^ about 10.4 million MTB‐infected persons developed TB (including 6.3 million new cases). Among these cases, approximately 1.674 million individuals died of TB.^2^ China ranked 3rd among the country with high TB burden, next to India and Indonesia. In 2016, 895 000 individuals were infected with MTB, while 51 800 persons died of TB. Although the incidence and mortality of TB gradually decreased through Directly Observed Therapy Short Course (DOTS) strategy, TB remained as a major public health problem due to the increasing number of HIV‐infected persons, drug‐resistant TB cases, and healthcare delay.^3^

The genotypes of MTB have proven to be a valuable tool in characteristics of strains, treatment effect of drug‐resistant TB, tracking transmission, predicting outbreaks and pathological properties, and evaluating the immune response.^4^, ^5^ IS6110‐RFLP is the first and classic method for genotyping MTB strains and is widely used since the early 1990s.^6^ Many methods have been invented based on PCR.^7^ Recently, the mycobacterial interspersed repetitive unit‐variable number tandem repeat (MIRU‐VNTR) method is the prevailing clinical technique because of its rapid, better repeatability, high‐throughput, and digitized analysis.^8^, ^9^ Among the different sets of MIRU‐VNTR loci described for genotyping MTB isolates,^10^, ^11^ a system based on 12, 15, and 24 loci is currently the most widely used in TB control systems in China.^9^, ^12^, ^13^ It aids in the elucidation of the phylogenetic relationships among clinical isolates and the identification of high‐risk groups that are susceptible to TB infection. Beijing genotype is associated with drug resistance, showing an increased drug resistance level. It possibly explains the wide distribution of the Beijing family.^14^ However, other studies did not show a relationship between drug resistance and strain clade.^15^

Yunnan belongs to the regions in China, where TB is prevalent. TB morbidity reached 6‰ in Yunnan population due to high incidence of various infectious diseases, large rural cohort, and limited medical condition.^16^ In addition, the genetic diversity of MTB was more plenty in Yunnan than in the other regions.^12^, ^17^ This study aims to investigate the genetic diversity and molecular epidemiology of MTB in the Yunnan population.

## 2 MATERIALS AND METHODS

### 2.1 MTB strain collection and drug susceptibility testing (DST) assay

A total of 430 samples of MTB strains were obtained from pulmonary TB patients from December 2013 to September 2017, who were diagnosed and treated by doctors in the Third People's Hospital of Kunming. The strains were cultured in Löwenstein‐Jensen (LJ) media. All pulmonary TB patients were from Yunnan Province. DST was performed in all MTB strains by using five first‐line drugs (streptomycin [SM], pyrazinamide [PZA], isoniazid [INH], rifampin [RIF], and ethambutol [EMB]), and part of the cases was reported in our previous study.^18^ Written informed consents conforming to the tenets of the Declaration of Helsinki were obtained from each participant prior to the study. This study was approved by the institutional review board of Kunming University of Science and Technology.

### 2.2 24‐locus MIRU‐VNTR genotyping

Genomic DNA was extracted from all MTB isolates by using the Bacteria DNA Kit (TIANamp, China) according to the manufacturer's instructions. Fragments of 24‐locus MIRU‐VNTR were amplified and genotyped as previously described.^10^ The PCR products were analyzed by using 3% agarose electrophoresis at 5 V/cm for 90 minutes. The 100‐bp DNA ladder and the amplicons of standard strain H37Rv were used as markers to evaluate the results. 5% of the genotyping results were randomly identified through direct sequencing.

### 2.3 Data analysis

The allelic diversity of the VNTR locus was calculated by using the formula *h = *1‐∑x_i_^2^ [n/(n −1)], where x_i_ is the allele frequency of locus, and n is the number of isolates. The *h* index could indirectly evaluate the heterogeneity of the locus in MTB strains. The Hunter‐Gaston discriminatory index (HGDI) was used to calculate the discriminatory power of each method.^19^ The VNTR loci were classified into three groups, as described by Sola et al,^20^ according to the HGDI. In brief, the VNTR loci were designated as high, moderate, and poor discriminatory at HGDI of >0.6, ⩾0.3 and ⩽0.6, and <0.3, respectively. The dendrogram was generated by using the unweighted pair group method with arithmetic mean (UPGMA). Beijing (ID: 3243/02 and 2351/02), LAM (ID: 4428/02, 4431/02, 7968/03, 8885/03, 4218/03, 4219/03, 4220/03, and 4221/03), T (ID: 5211/02, 5212/02, and 5213/02), Delhi/CAS (ID: 1805/02 and 7936/01), MANU2 (ID: 3215/01), and NEW‐1 (ID: 8870/03) family strains were used as reference strains to construct the genetic phylotree of MTB, which were downloaded from the MIRU‐VNTRplus web (http://www.miru-vntrplus.org). The clustering rate was calculated following the method of Zhao et al^9^ All data were analyzed through using the SPSS software package (Version 19.0), and statistical difference was considered at *P < *0.05.

## 3 RESULTS

### 3.1 Results of DST

A total of 74 MTB strains were identified as drug‐resistant TB through DST. Among which, 38 and 36 strains were multidrug‐ and single‐resistant TB strains, respectively. Multidrug‐resistant TB strains account for 51.4% of the drug‐resistant strains. Among the single‐resistant TB strains, the number of SM, PZA, INH, RIF, and EMB strains were 4 (4/430 = 0.93%), 6 (6/430 = 1.40%), 17 (17/430 = 3.95%), 4 (4/430 = 0.93%), and 5 (5/430 = 1.16%), respectively. The remaining 356 (356/430 = 82.79%) isolates were drug‐sensitive TB strains.

### 3.2 Comparison among 12‐, 15‐, and 24‐locus MIRU‐VNTR

**Figure 1:**
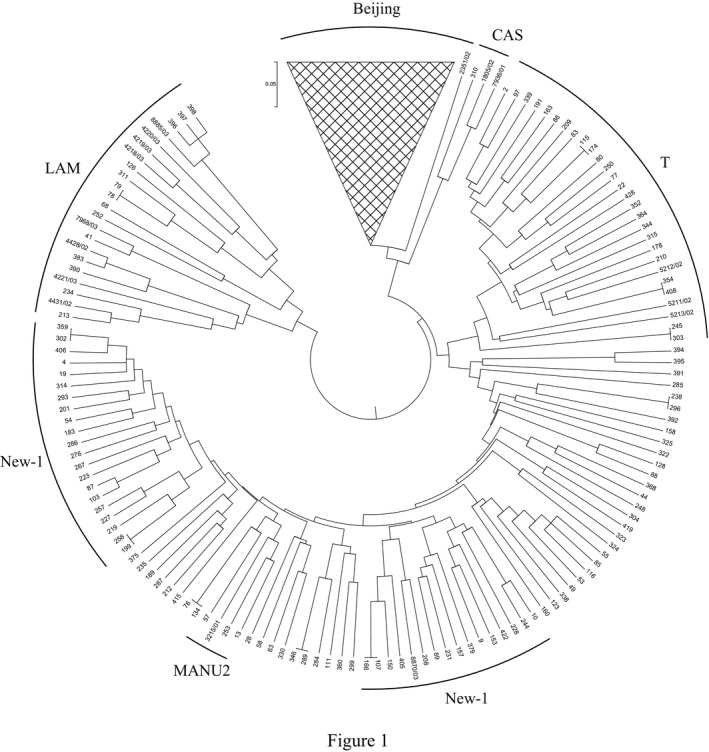
UPGMA tree of 430 clinical MTB strains based on 24‐locus MIRU‐VNTR by using the MIRU‐VNTRplus database. Sample number and family of each strain were noted in the figure

Considering that the genotyped 24 loci could cover all the loci of the 12‐ and 15‐locus MIRU‐VNTR, we first compared the HGDI of the 12‐, 15‐, and 24‐locus MIRU‐VNTR. The HGDI of the 15‐ (0.9996) and 24‐locus (0.9997) was slightly higher than that of 12‐locus (0.9928). A total of 287, 397, and 400 genotypes were obtained from 430 isolates by using 12‐, 15‐, and 24‐locus MIRU‐VNTR with clustering rate of 33.26%, 8.37%, and 6.98%, respectively (Table 1 and Figure 1). These results indicated the high allelic diversity of MTB strains determined by the 24‐locus MIRU‐VNTR. Compared with the 12‐locus MIRU‐VNTR, the number of unknown MTB strains in 15‐ and 24‐locus MIRU‐VNTR was significantly less (Table 2). Besides, the 15‐ and 24‐locus MIRU‐VNTR methods had improved the discrimination of Beijing, CAS, NEW‐1, and Unknown MTB strains. 24‐locus MIRU‐VNTR was used in further analysis of this study.

**Table 1:** Comparison of discriminatory power of 12‐, 15‐, and 24‐locus MIRU‐VNTR

Typing method	No. of genotypes	No. of unique genotypes	No. of clusters	No. of clustered isolates	Maximum no. of isolates in one cluster	Clustering rate (%)	HGDI
12‐locus	287	221	66	209	31	33.26	0.9928
15‐locus	397	365	32	65	3	8.37	0.9996
24‐locus	400	370	30	60	2	6.98	0.9997

HGDI means the Hunter‐Gaston discriminatory index.

**Table 2:** Lineages distribution through 12‐, 15‐, and 24‐locus MIRU‐VNTR

Lineage	12‐locus	15‐locus	24‐locus	*P* [Link]	*P* [Link]	*P* [Link]
Beijing	215 (50%)	305 (70.93%)	303 (70.47%)	0.001*	0.001*	0.881
T	29 (6.74%)	25 (5.81%)	25 (5.81%)	0.574	0.574	1.000
MANU2	2 (0.50%)	2 (0.50%)	3 (0.70%)	1.000	0.654	0.654
LAM	15 (3.50%)	14 (3.26%)	14 (3.26%)	0.850	0.850	1.000
CAS	12 (2.79%)	1 (0.23%)	1 (0.23%)	0.002*	0.002*	1.000
NEW‐1	2 (0.47%)	31 (7.21%)	36 (8.37%)	0.001*	0.001*	0.525
Unknown	155 (36%)	52 (12.06%)	48 (11.16%)	0.001*	0.001*	0.670

*P* value of < 0.05 was labeled as *.

Means *P* value was calculated between 12‐locus MIRU‐VNTR and 15‐locus MIRU‐VNTR.

Means *P* value was calculated between 12‐locus MIRU‐VNTR and 24‐locus MIRU‐VNTR.

Means *P* value was calculated between 15‐locus MIRU‐VNTR and 24‐locus MIRU‐VNTR.

### 3.3 Allelic diversity of 24 MIRU‐VNTR loci

5% MIRU‐VNTR genotypes were randomly identified by using direct sequencing. A total of 400 distinct genotypes were identified in 430 TB strains through 24‐locus MIRU‐VNTR analysis, containing 370 unique isolates (86.05%) and 30 (13.95%) pairs of isolates (Table 1). Beijing family was the most predominant lineage in Yunnan MTB strains (303/430 = 70.47%). Other lineages included T (25/430 = 5.81%), LAM (14/430 = 3.26%), MANU2 (3/430 = 0.7%), CAS (1/430 = 0.23%), NEW‐1 (36/430 = 8.37%), and unknown clades (48/430 = 11.16%) (Table 2).

The diversity of each allele significantly differed from 0.012 to 0.817 (Table 3). QUB11b and MIRU02 showed the highest and lowest allelic diversity, respectively. Among the loci, 9 (MIRU02, MIRU04, MIRU20, MIRU23, MIRU24, MIRU27, ETR‐C, Mtub29, and Mtub34), 8 (MIRU10, MIRU16, MIRU40, ETR‐A, ETR‐B, Mtub30, Mtub39, and QUB4156), and 7 (MIRU26, MIRU31, MIRU39, Mtub04, Mtub21, QUB11b, and QUB26) showed poor, moderate, and high diversity, respectively (Table 3). In comparison with the *h* value of each locus between Beijing and non‐Beijing family, most of the above‐mentioned loci showed significantly different diversity, thereby indicating their various discriminability (Table 4).

**Table 3:** Allelic diversity of 430 MTB strains by 24‐locus MIRU‐VNTR

Alias	Copy no. of repetitive units														*h* ^†^	diversity
	0	1	2	3	4	5	6	7	8	9	10	11	12	13		
MIRU 02	1	2	427												0.012	Poor
Mtub04		11	101	95	218	5									0.637	High
ETR‐C		1	6	15	402	6									0.122	Poor
MIRU 04	7	13	379	8	12	6	1	1	2					1	0.219	Poor
MIRU 40		26	58	308	28	6	4								0.459	Moderate
MIRU 10		20	134	263	11	1		1							0.525	Moderate
MIRU 16		11	53	281	79	6									0.522	Moderate
Mtub21		27	13	111	157	105	6		4	3	4				0.735	High
MIRU 20		7	422	1											0.034	Poor
QUB11b	1	9	20	75	68	65	122	62	7			1			0.817	High
ETR‐A		3	28	107	291	1									0.475	Moderate
Mtub29			6	11	393	17	1	2							0.160	Poor
Mtub30		2	126	7	291	4									0.455	Moderate
ETR‐B		93	336	1											0.341	Moderate
MIRU 23			3	4	7	390	23	3							0.172	Poor
MIRU 24	1	426	3												0.016	Poor
MIRU 26		9	6	10	19	93	58	149	69	14	1	2			0.784	High
MIRU 27		7	31	390	2										0.170	Poor
Mtub34		1	21	404	1	3									0.113	Poor
MIRU 31			18	96	29	258	23	4	2						0.580	High
Mtub39		25	28	282	83	12									0.523	Moderate
QUB26			6	6	11	6	30	83	237	37	8	6			0.644	High
QUB4156		9	246	28	142	3	2								0.558	Moderate
MIRU 39	6	41	195	178	10										0.612	High

*h* ^†^ represents the *h* value of allelic diversity of each locus. The VNTR loci were designated as high, moderate, and poor discriminatory at *h* of >0.6, ⩾0.3 and ⩽0.6, and <0.3, respectively.

**Table 4:** Allelic diversity of each locus in different MTB subgroups

Alias	Locus	Allelic diversity (*h*) for				
		Drug‐sensitive TB (n = 356)	MDR‐TB (n = 38)	SDR‐TB (n = 36)	Beijing family (n = 303)	Non‐Beijing (n = 127)
MIRU 02	154	0.008	0.026	0	0.003	0.023
Mtub04	424	0.646	0.467	0.652	0.543	0.676
ETR‐C	577	0.116	0.175	0.081	0.105	0.142
MIRU 04	580	0.222	0.220	0.133	0.147	0.182
MIRU 40	802	0.482	0.127	0.470	0.307	0.663
MIRU 10	960	0.527	0.538	0.411	0.367	0.529
MIRU 16	1644	0.535	0.407	0.463	0.486	0.557
Mtub21	1955	0.739	0.664	0.684	0.626	0.451
MIRU 20	2059	0.036	0	0.027	0.023	0.023
QUB11b	2163b	0.816	0.791	0.821	0.786	0.777
ETR‐A	2165	0.481	0.216	0.557	0.186	0.516
Mtub29	2347	0.152	0.216	0.132	0.147	0.184
Mtub30	2401	0.456	0.290	0.484	0.163	0.413
ETR‐B	2461	0.357	0.122	0.327	0.073	0.449
MIRU 23	2531	0.186	0	0.183	0.105	0.326
MIRU 24	2687	0.008	0.077	0	0.023	0
MIRU 26	2996	0.776	0.780	0.811	0.723	0.742
MIRU 27	3007	0.168	0.077	0.229	0.049	0.220
Mtub34	3171	0.105	0.122	0.132	0.091	0.169
MIRU 31	3192	0.584	0.459	0.583	0.379	0.600
Mtub39	3690	0.519	0.373	0.643	0.414	0.616
QUB26	4052	0.655	0.595	0.551	0.626	0.691
QUB4156	4156	0.548	0.626	0.516	0.607	0.221
MIRU 39	4348	0.607	0.626	0.597	0.591	0.384

### 3.4 Higher frequency of MDR‐TB strains in Beijing family

When we divided 430 MTB strains into three groups (drug‐sensitive TB, MDR‐TB, and SDR‐TB), the allelic diversity of each locus differed (Table 4). Consequently, the evaluation of each locus for allelic diversity varied. The statistic results showed significantly higher frequency of MDR‐TB in Beijing family than in non‐Beijing family (*P* = 0.032) (Table S1). However, no statistical difference in SDR‐TB frequency was identified between them.

## 4 DISCUSSION

TB is an ancient infectious disease, which remains as a global health problem. More than one million deaths from MTB infection is recorded worldwide every year.^2^ China experiences the highest TB and/or MDR‐TB burden in the world,^18^ which necessitates the prevention of MTB infection in Chinese population. MTB infection is one of the most serious infectious diseases in Yunnan, and the occurrence of drug‐resistant TB reached 37.91% in Yunnan MTB strains as reported by the Center for Disease Control and Prevention (CDC). Investigation on the genetic diversity of clinical MTB is a key way to control the prevalence of MTB in Yunnan Province, but several studies are performed in Yunnan MTB strains.^13^, ^21^

Here, we first calculated the HGDI of the 12‐, 15‐, and 24‐locus MIRU‐VNTR, and the results showed similar HGDI for the 15‐ and 24‐locus MIRU‐VNTR, which was higher than that in the 12‐locus MIRU‐VNTR. However, the clustering rate showed a noticeable decrease of 26.28% (12‐locus vs 24‐locus) and 24.89% (15‐locus vs 24‐locus) in the 12‐locus MIRU‐VNTR. In addition, the lineages of MTB strains showed significant difference from the analysis between 12‐locus and 15‐locus/24‐locus MIRU‐VNTR. Thus, the 24‐locus MIRU‐VNTR was used for further analysis. A highly different allelic diversity was identified in 430 MTB strains, and about 86.05% (370/430) of which showed unique genotype by using the 24‐locus MIRU‐VNTR. Moreover, the MTB strains had a relatively higher diversity in this study than that in Jiangsu strains (78.50%, 204/260),^22^ Tibet strains (44.29%, 229/517),^12^ and the strains reported (65.31%, 177/271) in Yunnan Province.^13^ Therefore, the 24‐locus MIRU‐VNTR analysis should be preferred in research and in clinical applications over the 12‐ or 15‐locus method.

Similar to the results of Chen et al,^13^ the Beijing family is the dominant lineage in Yunnan clinical MTB strains. However, the proportion of Beijing family is 55.7% and 70.47% in Chen et al and in this study (*P* < 0.01), respectively. The MIRU10 and MIRU31 loci showed high diversity between the Beijing and non‐Beijing family (Table S2). The difference in two studies might be due to the use of different sample sizes and genotyping methods (only using the 12‐locus VNTR method in Chen et al). Although the frequency of Beijing family in our study was similar to that in Sichuan (69.28%),^9^ Chongqing (66.7%),^14^ and Zhejiang (71.6%),^23^ the other TB lineage expressed minor difference. In comparison with other countries, the frequency of Beijing family in Yunnan Province was notably higher than that in Vietnam (35%),^24^ Myanmar (32%),^25^ India (8%),^26^ Iran (7.1%),^27^ and Pakistan (3%)^28^ and was similar as that in Russia (67.9%),^29^ Japan (73.8%),^11^ and Korea (80%).^30^ The transmission of MTB strains between Yunnan Province and Southeast Asia might be limited. However, a strain belonging to the CAS lineage was first identified in this study. The mainly prevalent region of the CAS family was India,^26^ which was also reported in Tibet,^12^ Xinjiang,^31^ Jiangsu,^22^ and Gansu^32^ in China. The transmission way of CAS MTB strain into Yunnan remains unclear. Many factors could cause the spread of TB disease,^31^ such as trade, population migration, and tourism. Thus, the CAS lineage in Yunnan MTB strains might have come from other Chinese provinces.

Among the 74 drug‐resistant TB isolates, 36 (48.65%, 36/74) strains were SDR‐TB. The rate of MDR‐TB in total TB was 8.84% (38/430), which was higher than that in the national survey in 2007.^33^ A significantly higher proportion of MDR‐TB strains was identified in Beijing family (12.56%, 54/430) than that of non‐Beijing family (4.65%, 20/430) (*P* = 0.032), but the SDR‐TB strains showed a similar ratio. This result was consistent with a previous study.^21^ The high frequency of MDR‐TB in Beijing family might explain its easy global distribution.^34^, ^35^ The analysis of epidemic characteristics in MDR‐TB strains will aid the prevention of TB infection in the Yunnan population.

In conclusion, MTB strains showed high genetic diversity in Yunnan, China, and Beijing family was the dominant lineage in both total MTB and MDR‐TB strains. Although a MTB strain belonging to the CAS lineage was identified in this study, we could not interpret its transmission way. Thus, the more molecular genetic characteristics of MTB strains should be further studied.
