# Supplementary material for: Genetic diversity and drug resistance of Mycobacterium tuberculosis in Yunnan, China
Source: J Clin Lab Anal. 2019 Mar 21;33(5):e22884. doi: 10.1002/jcla.22884 (PMC6595362; doi:10.1002/jcla.22884)
Supplement: Supplementary file 1 [file JCLA-33-e22884-s001.docx]

Table S1. Frequency of drug-resistant TB between Beijing and non-Beijing family

| Drug-resistant profiles | Total No. of isolates | No. (%) of isolates | | *P* value |
| --- | --- | --- | --- | --- |
|  |  | Beijing (n=303) | Non-Beijing (n=127) |  |
| MDR-TB | 38 | 33 (10.89%) | 5 (3.94%) | 0.032* |
| SM | 4 | 2 (0.66%) | 2 (1.57%) | 0.373 |
| PZA | 6 | 5 (1.65%) | 1 (0.79%) | 0.492 |
| INH | 17 | 9 (2.97%) | 8 (6.30%) | 0.123 |
| RIF | 4 | 3 (0.99%) | 1 (0.79%) | 0.843 |
| EMB | 5 | 2 (0.66%) | 3 (2.36%) | 0.139 |

Note: MDR-TB, multi-drug-resistant tuberculosis; SM, Streptomycin; PZA, Pirazinamide; INH, Isoniazid; RIF, Rifampicin; EMB, Ethambutol. *P* value of <0.05 was labeled as *.

Table S2. HGDI comparison of nine loci between Chen *et al.*^13^ and this study

| Alias | HGDI in Chen *et al.* | | | HGDI in this study | | |
| --- | --- | --- | --- | --- | --- | --- |
|  | All strains (n=271) | Beijing family (n=151) | Non-Beijing family (n=120) | All strains (n=430) | Beijing family (n=303) | Non-Beijing family (n=127) |
| Mtub04 | 0.647 | 0.397 | 0.604 | 0.637 | 0.547 | 0.684 |
| MIRU 40 | 0.476 | 0.243 | 0.639 | 0.459 | 0.310 | 0.671 |
| MIRU 10 | 0.570 | 0.162 | 0.274 | 0.525 | 0.370 | 0.537 |
| Mtub21 | 0.790 | 0.705 | 0.469 | 0.735 | 0.629 | 0.459 |
| QUB11b | 0.830 | 0.731 | 0.714 | 0.817 | 0.789 | 0.785 |
| MIRU 26 | 0.799 | 0.655 | 0.832 | 0.784 | 0.726 | 0.750 |
| MIRU 31 | 0.630 | 0.277 | 0.334 | 0.580 | 0.382 | 0.608 |
| QUB26 | 0.800 | 0.632 | 0.907 | 0.644 | 0.629 | 0.699 |
| QUB4156 | 0.533 | 0.625 | 0.228 | 0.558 | 0.610 | 0.229 |

Note: HGDI means the Hunter-Gaston discriminatory index.
